# Associations between Genomic Variants and Antifungal Susceptibilities in the Archived Global *Candida auris* Population

**DOI:** 10.3390/jof10010086

**Published:** 2024-01-22

**Authors:** Yue Wang, Jianping Xu

**Affiliations:** Department of Biology, McMaster University, Hamilton, ON L8S 4K1, Canada; wangy660@mcmaster.ca

**Keywords:** *Candida auris*, human fungal pathogen, genome-wide association study, antifungal susceptibility

## Abstract

*Candida auris* is a recently emerged human fungal pathogen that has posed a significant threat to public health. Since its first identification in 2009, this fungus has caused nosocomial infections in over 47 countries across all inhabited continents. As of May 2023, the whole-genome sequences of over 4000 strains have been reported and a diversity of mutations, including in genes known to be associated with drug resistance in other human fungal pathogens, have been described. Among them, 387 strains contained antifungal-susceptibility information for which different methods might be used depending on the drugs and/or investigators. In most reports on *C. auris* so far, the number of strains analyzed was very small, from one to a few dozen, and the statistical significance of the relationships between these genetic variants and their antifungal susceptibilities could not be assessed. In this study, we conducted genome-wide association studies on individual clades based on previously published *C. auris* isolates to investigate the statistical association between genomic variants and susceptibility differences to nine antifungal drugs belonging to four major drug categories: 5-fluorocytosine, amphotericin B, fluconazole, voriconazole, itraconazole, posaconazole, anidulafungin, caspofungin, and micafungin. Due to the small sample sizes for Clades II, V, and VI, this study only assessed Clades I, III, and IV. Our analyses revealed 15 single nucleotide polymorphisms (SNPs) in Clade I (10 in coding and 5 in noncoding regions), 24 SNPs in Clade III (11 in coding and 13 in noncoding regions), and 13 SNPs in clade IV (10 in coding and 3 in noncoding regions) as statistically significantly associated with susceptibility differences to one or more of the nine antifungal drugs. While four SNPs in genes encoding lanosterol 14-α-demethylase *(ERG11*) and the catalytic subunit of 1,3-beta-D-glucan synthase (*FKS1*) were shared between clades, including the experimentally confirmed Ser639Phe/Pro missense substitutions in *FKS1* for echinocandin resistance, most of the identified SNPs were clade specific, consistent with their recent independent origins. Interestingly, the majority of the antifungal resistance-associated SNPs were novel, and in genes and intergenic regions that have never been reported before as associated with antifungal resistance. While targeted study is needed to confirm the role of each novel SNP, the diverse mechanisms of drug resistance in *C. auris* revealed here indicate both challenges for infection control and opportunities for the development of novel antifungal drugs against this and other human fungal pathogens.

## 1. Introduction

*Candida auris* is an ascomycete fungus that has recently gained broad attention due to its pathogenicity, resistance to many antifungal drugs, and ability to cause nosocomial outbreaks. In October 2022, this species was classified as one of the four critical human fungal pathogens by the World Health Organization [1]. Although *C. auris* was first reported in 2009 [2], its earliest presence in clinical settings dates back to 1996 in South Korea [3]. Since *C. auris* was not widely reported by clinics until 2009, its sudden emergence has been widely debated, including potential linkages to global climate changes [4]. Indeed, this fungus was recently isolated from tropical wetlands [5], suggesting that global warming may have boosted its adaptation to high temperatures and stresses that are similar to the conditions in human bodies [6]. In addition, *C. auris* has been successfully isolated from the surfaces of stored apples, dogs, and other surfaces, indicating a potential risk of colonization or infection in individuals exposed to these sources [7,8].

Aside from its tolerance to high temperatures and environmental stresses, most strains of *C. auris* that have been reported so far are resistant to at least one common antifungal drug. Currently, only four classes of antifungal drugs are widely prescribed to treat fungal infections in clinics, which include azoles, polyenes, echinocandins, and antimetabolites, each with its unique mode of action. Azoles disrupt the ergosterol biosynthesis pathway and ergosterol serves as a vital component of the fungal cell membrane [9]. Polyenes exert their action by directly binding to ergosterol and disrupting the integrity of the cell membrane, as well as causing oxidative damage [10]. Echinocandins suppress beta-(1,3)-D-glucan synthase, which is an enzyme for the synthesis of a vital component of the fungal cell wall [11]. Antimetabolites prevent nucleotide acid synthesis [12]. Unlike other *Candida* species, *C. auris* has strains that are naturally resistant to multiple commonly used antifungal drugs. For example, a multi-center study on 350 clinical *C. auris* isolates in India revealed that 90% of the isolates were resistant against fluconazole, 15% against 5-fluorocytosine, 8% against amphotericin B, and 2% against echinocandins [13]. Remarkably, a quarter of these isolates exhibited resistance to multiple classes of antifungal drugs [13]. Indeed, many studies have explored the molecular bases of antifungal resistance in *C. auris*, including identifying mutations and/or differences in expressions of specific genes such as those encoding drug targets and efflux pumps. Specifically, the commonly reported mutations included those in *ERG* family genes and ATP binding cassette alterations which are related to resistance to azoles and polyenes, mutations in the genes *FCY1*, *FCY2*, and *FUR1*, which are related to 5-fluorocytosine resistance, and mutations in *FKS* family genes, which are linked to resistance to echinocandins [14]. However, though many mutations have been reported in strains of *C. auris*, their importance in natural populations remains largely unknown.

A genome-wide association study (GWAS) is a commonly used approach to identify genomic variants that are linked to individual differences in phenotypic traits in natural populations of an organism. A GWAS usually involves collecting genomic data from a large sample with a wide range of phenotypic trait values, identifying genomic variants such as single-nucleotide polymorphisms (SNPs) among individuals, and statistically testing the association between identified genomic variants and their trait values. The Genome Association and Prediction Integrated Tool (GAPIT) is a popular R package that offers a wide range of models with which to perform GWAS analysis. The two most frequently used methods are Fixed and Random Model Circulating Probability Unification (FarmCPU) [15] and the Bayesian-information and Linkage-disequilibrium Iteratively Nested Keyway (BLINK) method [16]. Both approaches are based on mixed linear models. FarmCPU uses a bin method as it assumes that quantitative trait-related nucleotides are evenly distributed across the genome. In contrast, the BLINK method drops this assumption and accounts for SNP interactions by taking linkage disequilibrium information into consideration.

Genomic analyses have classified strains of *C. auris* into six distinct clades, with each clade showing geography-biased distribution and clonal expansion [16,17,18,19,20]. The genomic divergence between different clades can range from tens of thousands to hundreds of thousands of SNPs [19,20]. To eliminate the effects of population structures for strains from different clades and to reduce false positives, we conducted GWAS analyses on individual clades to assess and identify antifungal-susceptibility-related single-nucleotide polymorphisms in populations of *C. auris*.

## 2. Materials and Methods

### 2.1. Data Collection

As of 18 May 2023, the National Center for Biotechnology Information (NCBI) database contains a total of 4304 *C. auris* isolates with paired-end genomic data available. We performed a search of the literature to retrieve the corresponding minimum inhibitory concentrations (MIC) against antifungal drugs for these isolates from their corresponding publications. The distribution of MICs for each drug in each clade was visualized using ggplot2 [20] in the form of histograms. The raw paired-end genome data for all isolates with MIC data for any antifungal drugs were retrieved from the NCBI database for subsequent analyses.

### 2.2. Genomic Variant Calling and Annotation

In total, 387 isolates with whole-genome sequence data were found to also contain antifungal drug-susceptibility information in their corresponding metadata and/or associated publications. These isolates belonged to five clades, with 152 isolates in Clade I, 12 in clade II, 119 in clade III, 99 in clade IV, and 5 in clade V. Recently, 3 genetically divergent strains of *C. auris* were reported in Singapore, and were assigned to a new clade, Clade VI [19]. Due to the small sample sizes in Clades II, V, and VI, this study will focus on Clades I, III, and IV for its GWAS.

Genomic data of samples from Clades I, III, and IV with available MIC information were obtained from the NCBI database. Strains B8441 (I; GCA_002759435.2), B11221 (III; GCA_002775015.1), and B11245 (IV; GCA_008275145.1) were selected as reference genomes for their respective clades, due to the high level of genome annotation information for these three strains. For strains B8441 and B11221, MIC data were only available for six drugs while, for strain B11245, the MIC data were available for all nine antifungal drugs analyzed here (Table S1).

For strains within each clade, their whole genome SNPs were identified and filtered using the Northern Arizona SNP Pipeline (NASP) v1.12 [21]. Briefly, raw reads of samples were adapter and quality trimmed using Trimmomatic v0.39 [22]. Then, trimmed reads were aligned to the selected reference genome for their respective clades. Next, genomic variants were called using GATK UnifiedGenotyper [23]. Lastly, SNPs were filtered out if they failed to meet any of the following criteria: (i) located in unduplicated regions of the reference genomes; (ii) with a read depth no lower than 10; (iii) with a minimum read proportion of 0.9.

The SNPs and their effects were annotated and predicted using SnpEff [24]. The gene homology and function annotation for Clade I were obtained from the Candida Genome Database [25]. SNPs and genes of Clades III and IV were annotated by blasting the sequences containing the SNPs with the Clade I reference strain B8441.

### 2.3. SNPs in Antifungal-Related Genes for Individual Clades

Mutations and expression differences in 19 genes have been reported as related to antifungal resistance in *Candida* species [13]. These 19 genes are *FUR1*, *TAC1B*, *ERG3*, *ERG4*, *ERG5*, *ERG11*, *CDR1*, *MDR1*, *MSH2*, *PMS1*, *PDR1*, *HOG1*, *STE6*, *YMC1*, *MLH1*, *CDR2*, *CDR4*, *FKS1*, and *FKS2*. In this study, we performed SNP screening for these genes within each clade of *C. auris* and explored their relationships to MIC differences among strains across all clades.

### 2.4. Genome-Wide Association Study

Genome-wide association analysis was performed on each of the 9 antifungal drugs for each clade using both FarmCPU and BLINK from the GAPIT package [26]. Among relevant strains and studies, the format of antifungal drug susceptibility reporting varied widely. For our GWAS, we standardized the variable formats as follows. If an MIC range (instead of a specific MIC value) against a certain antifungal drug was reported for a strain, the strain was assigned to an adjusted MIC value for our analyses. Specifically, if the MIC was “>the maximum value” or “<the minimum value” for a certain drug, the strain was assigned an adjusted value of “the maximum value” plus one unit or “the minimum value” minus one unit, either −1, −0.1, or −0.01, depending on the number of digits present in the value. For instance, a strain with a reported MIC > 256 mg/L would be assigned a value of 257 mg/L, and a strain with a reported MIC < 0.06 mg/L was assigned 0.05 mg/L. In cases where the MIC was a closed interval, the mean value was assigned to those samples. In the following scenarios, samples were excluded for analyses. For example, in a clade, if strain A has an MIC for drug1 that is greater than v1, but v1 is not the maximum MIC value for drug1 in this clade, then strain A will be excluded from the analysis for that drug. Similarly, if strain B has an MIC for drug1 that is less than v2, but v2 is not the minimum MIC value for that drug, strain B will also be excluded. After MIC adjustments and filtering, we determined the final number of samples from each clade that would be used for GWAS analysis. Since antifungal testing based on microbroth dilution, the gold standard in this field, has been conducted based on a two-fold dilution of antimycotic agents, we also considered standardizing the MICs in a log scale. Specifically, in this alternative test, an MIC of 512 mg/L was assigned to strains with an MIC > 256 mg/L, and an MIC of 0.03 mg/L was assigned to strains with an MIC < 0.06 mg/L. This alternative test was applied to a GWAS of FLU for Clades I and III, and a GWAS of 5FC for Clade III.

The genomic variants were treated as the independent variables and the minimum allele frequencies were set as 0.01 for each analysis. Principal components of genomic variants were included as covariates to account for population structure, and the optimum number was determined based on a QQ plot for individual analyses. A GWAS was considered validated if the overall distribution of observed *p*-values, or at least most observed *p*-values, showed no deviation from the expected *p*-values, i.e., the points on the QQ plot align to the diagonal line (with a tail). The statistical significance of SNPs in relation to antifungal-susceptibility was assessed using false discovery rate (FDR) adjusted *p*-value.

### 2.5. Analysis of SNPs Associated with Antifungal-Susceptibility

For the Clade I population, genes harboring significant SNPs, identified above, were annotated according to the *Candida* genome database, which provides comprehensive information on the genes and genomes of Clade I reference B8441. For the populations of Clades III and IV, SNPs and genes were compared to the B8441 genome using BLAST [27] to obtain their annotation and function details. As for intergenic SNPs, four gamete tests and linkage disequilibrium tests were performed as described previously [28] to examine whether they are linked to other identified significant SNPs. To gain insights into the potential roles of these SNPs in antifungal-susceptibility, we carried out Gene Ontology (GO) enrichment analysis on the genes flanking the intergenic SNPs using Fungifun v2.2.8 to determine if they were linked to other significant SNPs [29].

## 3. Results

### 3.1. MIC Distribution

**Figure 1:**
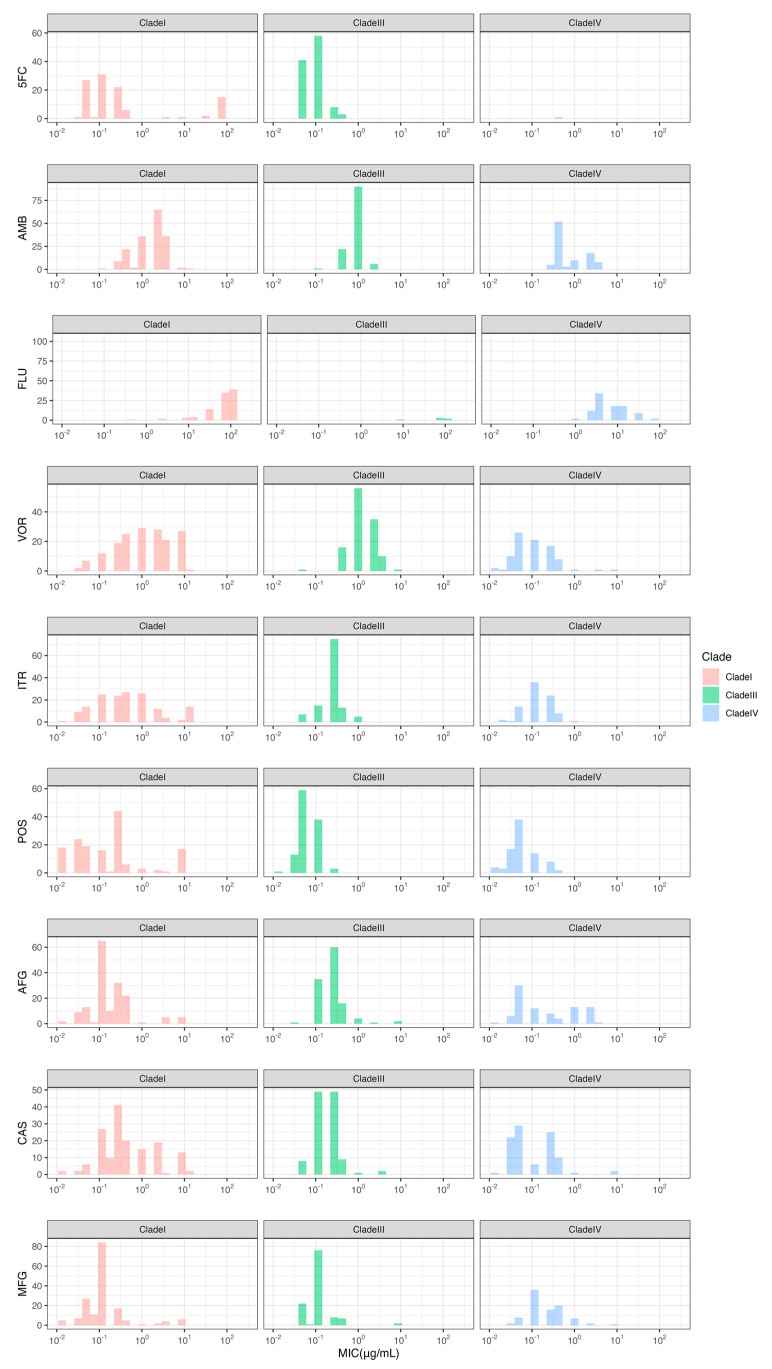
Histograms depicting the distributions of MICs (μg/mL) among strains for each clade against different drugs. Y-axis depicts the counts while x-axis depicts the MIC values, with 10^−2^, 10^−1^, 10^0^, 10^1^, and 10^2^ on the x-axis being equivalent to 0.01, 0.1, 1, 10, and 100 μg/mL.

Collectively, MIC values were obtained for 387 strains, consisting of 152 Clade I, 12 Clade II, 119 Clade III, 99 Clade IV, and 5 Clade V strains (Table S1). These strains come from 22 countries across six continents, including 189 from Asia, 82 from South America, 49 from North America, 47 from Europe, 11 from Oceania, 7 from Africa, and 2 with an unknown country of origin. Each of these strains contains an MIC value against at least one of the following nine antifungal drugs: 5-fluorocytosine (5FC), amphotericin B (AMB), fluconazole (FLU), voriconazole (VOR), itraconazole (ITR), posaconazole (POS), anidulafungin (AFG), caspofungin (CAS), and micafungin (MCF). Due to the limited number of strains from Clades II and V, data analysis was exclusively carried out on the remaining three clades. In total, the MICs of nine antifungal drugs were collected for Clades I, III, and IV (Table 1), and their distributions for each clade are shown in Figure 1.

### 3.2. Variant Calling and SNPs in Known Antifungal Resistance-Related Genes

Overall, the Clade I, III, and IV populations had 983, 1687, and 1031 genomic loci with SNPs as compared to their respective reference genomes. Among these genes, 19 genes have been reported to be associated with antifungal resistance in *Candida* species. We first specifically investigated the potential associations of SNPs in these 19 genes with antifungal-susceptibility differences within each clade. Overall, we identified 10, 8, and 9 SNPs in these 19 genes in the Clade I, III, and IV subpopulations, respectively, as being significantly associated with antifungal-susceptibility differences. The details about these SNPs are shown in Table S2. We compared the SNPs among the three clades and found four SNPs in the genes *EGR11* and *FKS1* that were shared between two of the three clades (Table 2). All four of these SNPs resulted in amino acid substitutions.

### 3.3. Clade I Genome-Wide Association Study

GWAS analysis was performed on Clade I strains to investigate genomic variants associated with antifungal susceptibility. Various numbers of principal components were examined to adjust the population structure. Except for 5FC, GWAS analyses for the rest of the examined antifungal drugs generated optimal QQplots. However, no significant SNPs were identified as being linked to ITR, VOR, or POS susceptibility differences in strains in Clade I of *C. auris*.

**Figure 2:**
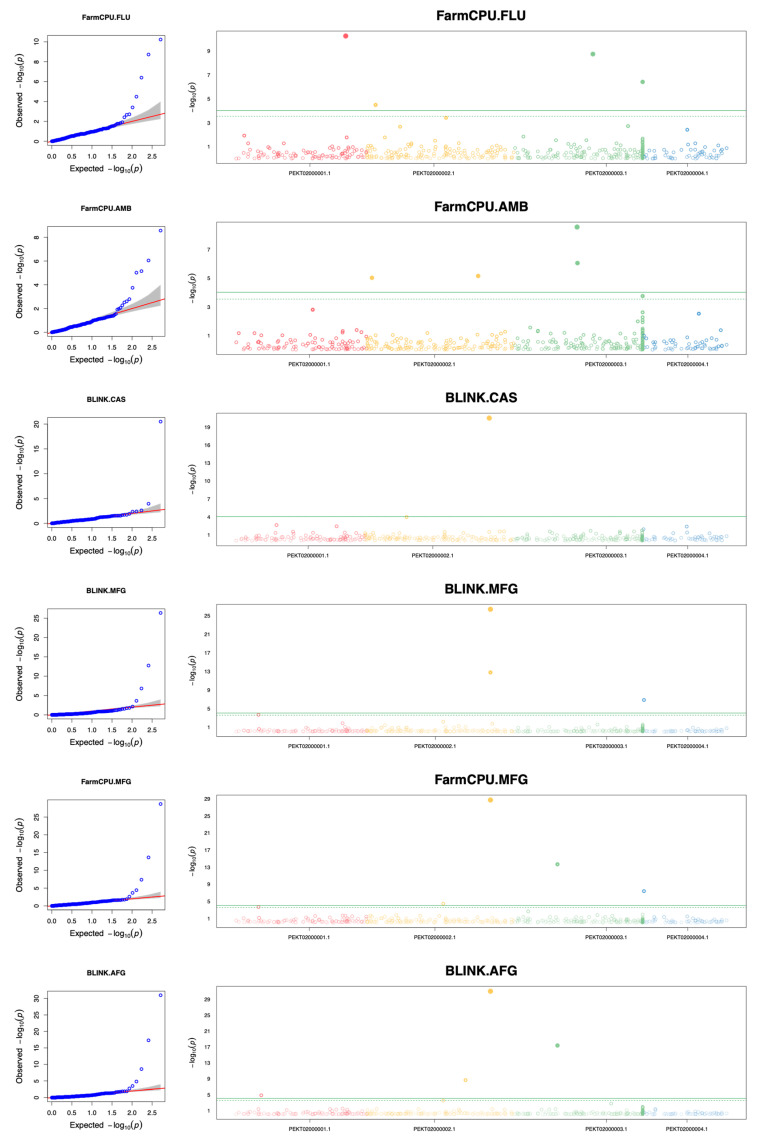
QQ plots and Manhattan plots showing genome-wide SNPs associated with antifungal-susceptibility differences among strains within Clade I. The left panel displays the QQ plots for five GWAS analyses, while the right panel presents the Manhattan plots. Plots are arranged from top to bottom in the following order: FLU, AMB, CAS, MFG, and AFG. The QQ plots display the expected −log10 (*p*-value) on the X-axis and the observed −log10 (*p*-value) on the Y-axis. The Manhattan plots are depicted with scaffold position on the X-axis and the −log10 (*p*-value) on the Y-axis. The significant *p*-value threshold for the SNPs is represented by green lines on the Manhattan plots.

We identified significant associations between genetic variants and susceptibility differences for the antifungals FLU, AMB, CAS, MFG, and AFG. The findings of the five GWAS analyses are summarized in Figure 2, which includes both a QQ Plot and Manhattan plot for each antifungal drug. Details of significant SNPs are presented in Table 3, showing the details of SNPs associated with differences in antifungal-susceptibility, including the antifungal agent, SNP ID, minor allele frequency, number of strains included in each GWAS analysis, adjusted *p*-value, effect of the presence of the SNPs on the MIC value, parameters for the GWAS analysis, mutation type, and the gene containing the SNP and its ortholog in *C. albicans*. Specifically, we observed putative associations of four SNPs with FLU, four with AMB, one with CAS, five with MFG, and four with AFG susceptibility differences (Table 3).

For the four SNPs associated with FLU susceptibility differences, three were located in intergenic regions, at the nucleotide sites PEKT02000001.1_897129, PEKT02000003.1_642734, and PEKT02000003.1_1045994. The fourth SNP, at site PEKT02000002.1_75394, introduced a synonymous mutation in the gene *B9J08_000534*. The orthologs of this gene have chromatin binding activity. Of note, the effect values revealed that the three intergenic SNPs had higher impacts on the FLU MICs than the fourth SNP. In detail, compared to the reference genome B8441, the PEKT02000001.1_897129 SNP had a negative effect on the FLU MIC, with its presence associated with a decrease in the MIC by 101.83 μg/mL. This SNP was located 179 nucleotides upstream of the gene *B9J08_000434*, whose orthologs have ATP/ADP antiporter activity. In contrast, the remaining three SNPs were all associated with an increased FLU MIC, with their effect values being 188.652, 201.408, and 65.982 (µg/mL), respectively.

Four SNPs were associated with AMB susceptibility differences, including two synonymous and two missense mutations. Specifically, SNPs at the sites PEKT02000003.1_514453 and PEKT02000003.1_517475 resulted in Ala224Ala and His283His synonymous mutations in the genes *B9J08_001302* and *B9J08_001303*, respectively, with both mutations having negative effects on AMB MICs. Of the two missense mutations, Ser489Pro in the gene *B9J08_000923* was associated with an increased AMB MIC, whereas the mutation Ile343Thr in *B9J08_000517* had a negative impact. The ortholog of *B9J08_000923* in *C. albicans*, *SWC4*, encodes a subunit of the NuA4 histone acetyltransferase complex, which is involved in DNA repair, chromatin remodeling, chromosome organization, etc. On the other hand, *B9J08_000517* is the ortholog of *C. albicans FET31*. *FET31* encodes a multicopper oxidase and is known to contribute to antifungal repression [30] and biofilm induction [31].

With respect to the three echinocandins, a single SNP at the site PEKT02000002.1_1006625 was found to be associated with a CAS susceptibility difference. This SNP converts serine in the reference strain to proline at position 639 of the gene that encodes beta-1,3-glucan synthase (*FKS1*). Position 639 is part of the catalytic subunit of Fks1p, and the mutation resulted in an increase in the CAS MIC by 7.42 μg/mL. The same SNP was also identified as increasing MICs for MFG. In addition, four other SNPs showed a significant association with MFG susceptibility differences. Among these four, two (at the sites PEKT02000002.1_1006624 and PEKT02000003.1_355435), that caused Ser639Phe mutations in Fks1p and an earlier stop codon in Fcr1p, were also found to be associated with AFG susceptibility. One significant SNP, at the position PEKT02000004.1_9898, was a synonymous mutation located in the gene *B9J08_001531*, the ortholog of which encodes a putative GPI-anchor [32]. The last SNP associated with an AFG susceptibility difference was in an intergenic region. Among the four SNPs associated with AFG MICs, two (at the sites PEKT02000002.1_1006624 and PEKT02000003.1_355435) were also related to MFG susceptibility, while the remaining two (at the sites PEKT02000002.1_804575 and PEKT02000001.1_212518) were specific to AFG susceptibility differences. The former introduced a synonymous mutation in the gene *B9J08_000876*, and the latter was an intergenic SNP.

Interestingly, the SNP at the site PEKT02000003.1_355435 was significantly associated with MIC differences for both MFG and AFG, with the mutant allele having reduced MIC values. This SNP resulted in a premature stop codon in the gene *B9J08_001232*, a homolog of *FCR1* and a zinc cluster transcription factor in *C. albicans*. Of note, *FCR1* has been found to repress azole and brefeldin resistance [33,34]. Additionally, the SNP at the site PEKT02000002.1_1006624 was identified to significantly increase MICs against both MFG and AFG. It caused a serine-to-phenylalanine/tyrosine mutation at position 639 in the product of gene *B9J08_000964*, which is known as 1,3-beta-D-glucan synthase subunit *Fks1p*, an essential enzyme for the synthesis of the main component of the fungal cell wall. The mutant allele showed increased echinocandin resistance in a murine model of infection [35]. Another SNP, at the site PEKT02000004.1_9898, was found to have a minor positive effect on AFG and MFG MICs. This SNP resulted in a synonymous variant in an uncharacterized gene, *B9J08_001531*. The homolog of this gene in *C. albicans* is *IFF4*, which encodes an adhesin-like cell surface protein [32]. The other synonymous variant that contributes to increased AFG MICs was the SNP at the site PEKT02000002.1_804575 in the gene *B9J08_000876*. This gene’s ortholog, *GWT1*, is involved in the biosynthesis of glycosylated proteins. A previous study showed that this gene had the potential to be a drug target in *C. albicans* and *Aspergillus fumigatus* [36].

### 3.4. Clade III Genome-Wide Association Study

**Figure 3:**
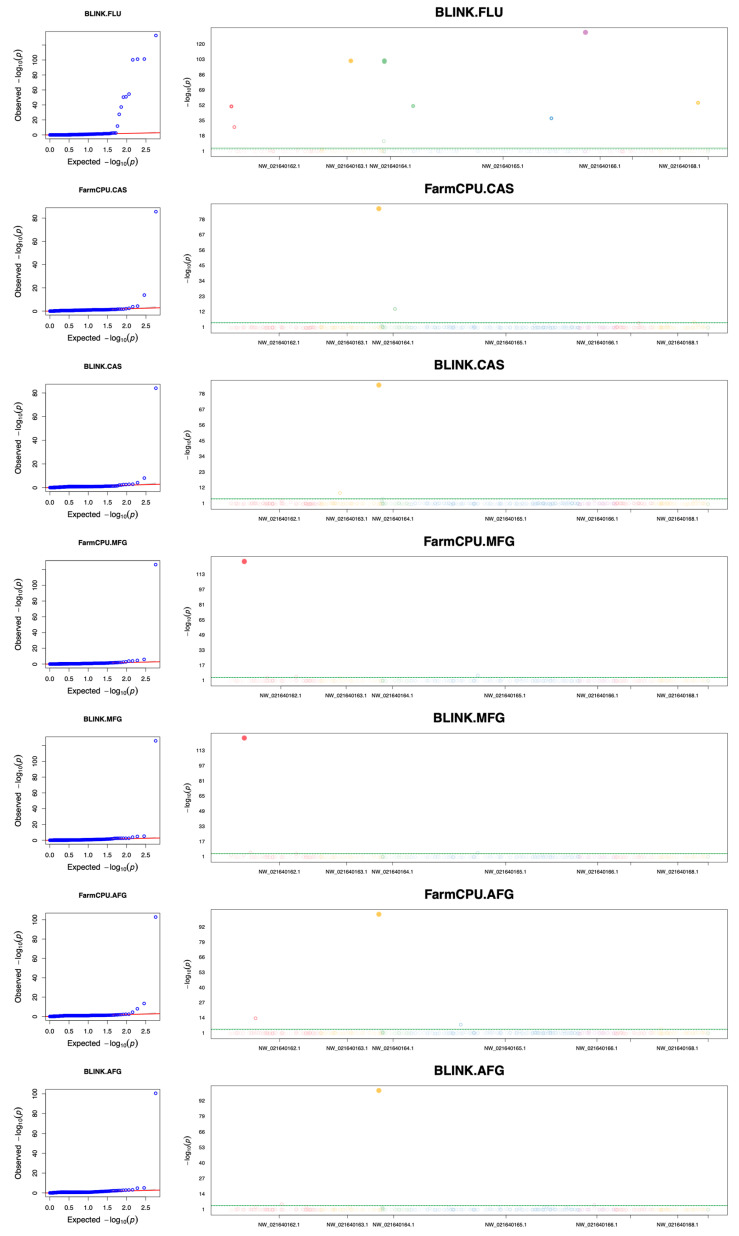
QQ plots and Manhattan plots showing genome-wide SNPs associated with antifungal-susceptibility differences among strains within Clade III. The left panel displays the QQ plots for five GWAS analyses, while the right panel presents the Manhattan plots. Plots are arranged from top to bottom in the following order: FLU, CAS (FarmCPU; BLINK), MFG (FarmCPU; BLINK), and AFG (FarmCPU; BLINK). The QQ plots display the expected −log10 (*p*-value) on the X-axis and the observed −log10 (*p*-value) on the Y-axis. The Manhattan plots are depicted with scaffold position on the X-axis and the −log10 (*p*-value) on the Y-axis. The significant *p*-value threshold for the SNPs is represented by green lines on the Manhattan plots.

GWAS analysis was performed for susceptibility differences among strains in nine antifungal drugs in Clade III. Significant associations were observed for four of these nine drugs, FLU, CAS, MFG, and AFG (Figure 3). Both the BLINK and FarmCPU methods identified several SNPs linked to echinocandins susceptibility. However, due to discrepancies in the results obtained from these two methods, only significant SNPs identified by both approaches were retained for further analysis (Table 4).

We observed 10 SNPs which showed significant associations with FLU susceptibility differences among strains within Clade III. Out of these 10 SNPs, two resulted in missense mutations, another two led to synonymous mutations, and the remaining six resulted in intergenic regions. All of the variants that were located in coding regions were positively related to FLU MICs except for the SNP at site NW_021640165.1_3471968, which altered glycine to valine at position 756 of B9J08_003726p. The ortholog of the gene *B9J08_003726* in *C. albicans* is *C2_04360W_A*, which encodes a putative protein kinase involved in the stress response [31,37]. The other missense SNP led to a conversion from alanine to serine at position 42 of B9J08_000558p. This gene has been predicted to be involved in transmembrane transport and an integral component of membrane localization [25]. Apart from the two missense variants, two synonymous variants in the genes *B9J08_004201* and *B9J08_000516* contributed to increased FLU MICs. Orthologs of the former gene are known to be involved in intracellular membrane transport and the regulation of GTPase activity, while orthologs of the latter gene exhibit phospholipase A2 activity and play a role in cardiolipin acyl-chain remodeling [25].

With respect to the three echinocandins, 15 SNPs were identified as having significant associations with MIC differences among strains. GWAS analysis for CAS MIC differences identified five SNPs, among which the SNP at site NW_021640163.1_1501688 was found to be positively associated with both AFG and CAS tolerance. This SNP introduced a synonymous mutation in *B9J08_001552*, a gene with an undetermined function. Meanwhile, a missense variant, at site NW_021640164.1_366204, was solely identified as being associated with CAS using FarmCPU, but with a weak effect. This variant converted arginine to serine at position 221 in B9J08_002399p. Its ortholog in *C. albicans* is Sup35p, a translation factor eRF3 [38]. Another variant with weak association is the SNP at site NW_021640168.1_1068005 in the gene *B9J08_005386*, which caused a premature stop codon. *B9J08_005386* was an uncharacterized gene but its protein product was predicted to have zinc ion binding activity [25]. On the other hand, a missense variant, at site NW_021640163.1_505104, was found to be associated with CAS susceptibility using BLINK. This SNP resulted in a Tyr236His mutation in the product of the gene *B9J08_001308*. Its ortholog *EIP1* encodes a separase-binding protein in *C. albicans* and has been implicated in regulating the cell wall integrity, filamentation, and response to antifungals in *C. albicans* [39].

Four SNPs were associated with MFG susceptibility differences among strains in Clade III, and two of them were detected by both the BLINK and FarmCPU methods. One of these two SNPs, at site NW_021640165.1_1713220, caused a missense mutation, changing the amino acid aspartic acid to glutamic acid in the product of the gene *B9J08_002877*. The orthologs of this gene have tubulin binding activity [25].

A missense variant and a synonymous variant were identified to positively contribute to AFG tolerance. The missense SNP at NW_021640162.1_692140 triggered a Ser174Cys mutation in the protein product of the gene *B9J08_000805*. Its ortholog in *C. albicans* is *ZCF19*, a predicted Zn(II)2Cys6 transcription factor [40]. Interestingly, the synonymous variant at site NW_021640163.1_1501688, related to AFG susceptibility difference, was also associated with CAS susceptibility difference.

### 3.5. Clade IV Genome-Wide Association Study

**Figure 4:**
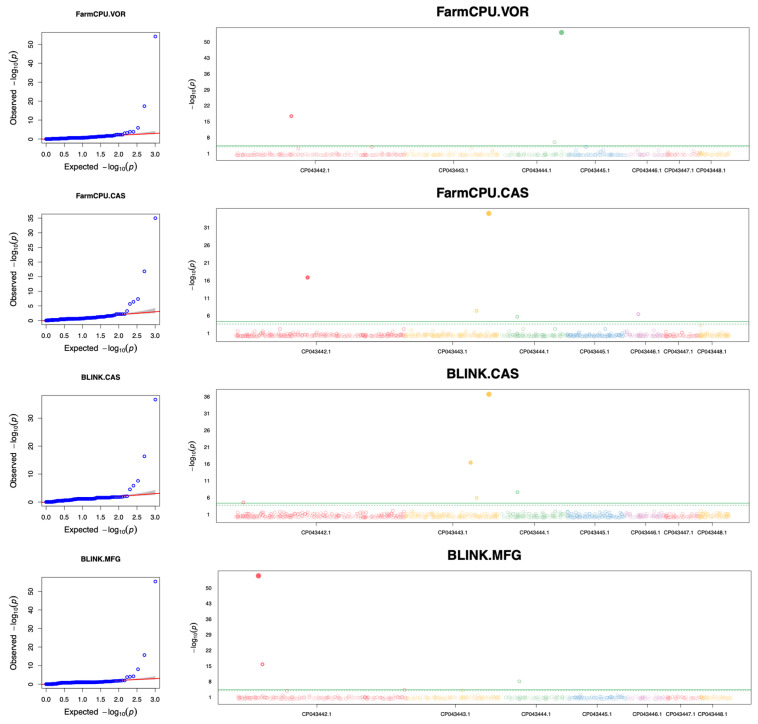
QQ plots and Manhattan plots showing genome-wide SNPs associated with antifungal-susceptibility differences among strains within Clade IV. The left panel displays the QQ plots for three GWAS analyses, while the right panel presents the Manhattan plots. Plots are arranged from top to bottom in the following order: VOR, CAS (FarmCPU; BLINK), and MFG. The QQ plots display the expected −log10 (*p*-value) on the X-axis and the observed −log10 (*p*-value) on the Y-axis. The Manhattan plots are depicted with scaffold position on the X-axis and the −log10 (*p*-value) on the Y-axis. The significant *p*-value threshold for the SNPs is represented by green lines on the Manhattan plots.

The associations between genome-wide SNPs and susceptibility differences among strains in Clade IV were assessed for eight antifungal drugs. Significant associations between SNPs and MIC differences were identified for three of the eight drugs. These three drugs are VOR, CAS, and MFG, among which CAS-associated SNPs were identified by both methods (Figure 4). The details of the significant SNPs are listed in Table 5.

Three SNPs were found to be associated with VOR susceptibility differences in Clade IV and all three were missense mutations. In particular, the SNP at site CP043444.1_1566757 resulted in a Phe132Tyr mutation in the gene *ERG11*, which encodes lanosterol 14-alpha-demethylase, an essential enzyme for ergosterol biosynthesis. This mutation has been frequently reported for FLU-resistant *C. auris* strains [13,41,42]. However, the other two missense variants have not been reported before as being associated with antifungal-susceptibility. One of these two variants, at nucleotide site CP043442.1_1367963, led to a Ser1443Leu mutation in the gene *B9J08_003473.* While this gene has not been fully characterized, its orthologs are known to have diverse roles and activities, such as binding to signal sequences and participating in different transport processes [25]. The second variant identified as associated with VOR susceptibility difference is at site CP043444.1_1393470, which caused a Asn39Ile mutation in the gene *B9J08_001368*. Its orthologs show glycerone kinase activity and affect the cellular response to toxic substances [25].

Five SNPs each were shown to be associated with CAS susceptibility differences by the FarmCPU and BLINK methods. Among the five SNPs, three were detected by both methods. They are an intergenic variant at site CP043443.1_1796780, a missense variant at site CP043443.1_2100709, and a stop-gained variant at site CP043444.1_498630. The variant at site CP043443.1_2100709 resulted in a replacement of serine with proline at position 639 in Fks1p. This mutation has been reported to be exclusively present in strains that are not susceptible to echinocandins [43,44,45]. On the other hand, FarmCPU identified two extra missense variants. One is at site CP043442.1_1778999, converting phenylalanine to leucine at position 1098 in the protein product of the gene *B9J08_003281*. Its orthologs have protein kinase activity. The other one, at site CP043446.1_343550 in the gene *B9J08_004612,* led to a Pro163Leu mutation. Orthologs of *B9J08_004612* exhibit mRNA binding activity [46].

For susceptibility differences to MFG, three genomic variants were identified as significantly associated. Among them, an SNP at site CP043444.1_498630, causing a premature stop, was also associated with CAS susceptibility. The remaining two were missense variants, at sites CP043442.1_514989 and CP043442.1_613136. Mutations at these two sites decrease the MFG tolerance. The SNP at site CP043442.1_514989 is located in the gene *B9J08_002701*, and its ortholog in *C. albicans* is associated with GTP binding, GTPase activity, and ribosome binding activity. The SNP at site CP043442.1_613136 is located in the gene *B9J08_003827*, whose ortholog has been predicted to be involved in ascospore formation and ascospore wall assembly [25].

### 3.6. Noncoding SNPs Associated with Antifungal-susceptibility Differences

Overall, we identified a total of 20 noncoding SNPs associated with antifungal-susceptibility differences (Table 6). Noncoding SNPs located in gene regulatory elements usually have impacts on the gene’s expression, thereby affecting traits of interest. However, it is also possible that their statistical association with drug resistance was due either to their tight linkage with drug resistance-associated genetic variants in coding regions or to synergistic interactions with each other. To identify the potential relationships between intergenic SNPs with each other and with drug resistance-associated genetic variants in coding regions, we performed four-gamete tests and linkage disequilibrium tests between significant antifungal-susceptibility-associated SNPs in the noncoding and coding sequences. The four-gamete tests and linkage disequilibrium tests on individual clades uncovered that the noncoding SNP at site PEKT02000003.1_642734 of Clade I was in linkage disequilibrium with the coding SNP at site PEKT02000002.1_1006624, which has been demonstrated in a previous study to confer echinocandin resistance in *C. auris* [47]. Of note, this noncoding SNP had a positive effect on FLU MICs. The other two intergenic SNPs, at sites PEKT02000001.1_897129 and PEKT02000003.1_1045994, were in significant linkage disequilibrium in Clade I. Similarly, two intergenic SNPs in Clade III were also in significant linkage disequilibrium. The significant associations between these three pairs of SNPs suggest that either they interact with each other to influence antifungal-susceptibility differences or that one SNP in each pair was hitchhiking the other SNP.

The linkage disequilibrium analysis of significant SNPs identified by GWAS analysis aimed to examine the association between SNPs in noncoding regions versus coding regions. The results showed that one SNP in a noncoding region, i.e., PEKT02000003.1_642734, was statistically linked with PEKT02000002.1_1006624, which was in a coding sequence (Table 6). For this and 19 other noncoding SNPs that are significantly associated with antifungal-susceptibility differences, we hypothesized that they potentially affected the expression of adjacent genes to impact antifungal-susceptibility (Table S3). To infer the functions and roles of genes adjacent to these 20 intergenic SNPs, we carried out GO enrichment analysis. Several SNPs were located at the end of the chromosome where there are no downstream genes. Therefore, 30 genes were confirmed to be located upstream or downstream of these SNPs. Because *C. auris* annotation has not been incorporated in Fungifun, we performed GO enrichment analysis on their orthologs in *C. albicans* SC5314. The orthologs of only 28 genes were successfully retrieved from the Candida genome database (the other two genes do not have orthologs in *C. albicans*). Fourteen of the 28 genes were found in several significantly enriched categories using Fisher’s exact test with a false discovery rate (FDR) adjusted criterion of 0.05 (Table S4). These categories include metabolic processes, regulations and response, transport process, biosynthetic processes, and cellular localization. For instance, the gene *B9J08_000434*, located 180 bp downstream of the noncoding SNP PEKT02000001.1_897129, is known to be involved in several essential cellular processes that potentially affect pathogenicity, such as anaerobic respiration, the aerobic glycerol catabolic process, nucleotide transmembrane transport, ion transmembrane transport, and ADP transport.

### 3.7. Alternative GWAS Analyses for Three Groups

We conducted additional GWAS analysis for FLU susceptibility for Clades I and III, and for 5FC susceptibility for Clade III, using MIC data standardized in a log scale. Among the three additional tests, we were able to generate an optimal QQ plot for only FLU susceptibility for Clade I (Figure S1). In this new optimal QQ plot, eight SNPs were found to be associated with FLU susceptibility (Table S5). Among these eight SNPs, three introduced missense mutations, two resulted in synonymous mutations, and the other three occurred in noncoding regions. One of these eight SNPs, an intergenic SNP (G->T) at site PEKT02000001.1_897129, was also identified in the original FLU GWAS for Clade I. Interestingly, a new SNP identified here at site PEKT02000002.1_906944 (Ser489Pro in the gene *SWC4*) was also linked to high AMB MICs in original GWAS analyses in Clade I.

## 4. Discussion

*C. auris* has recently drawn a lot of attention due to its clinical significance. The treatment of infections caused by *C. auris* faces great challenges due to the multidrug resistance of many strains, its resistance to environmental stresses, and, notably, the associated high mortality rates in infections. Hence, there is an urgent need to elucidate the underlying mechanisms of resistance and to develop biomarkers for the rapid diagnosis of drug resistance and drug targets [48].

In this study, we investigated the relationships between genomic SNPs and the antifungal-susceptibility differences in *C. auris.* To the best of our knowledge, this study encompasses all documented *C. auris* samples containing publicly available genomic data and MIC profiles up to May 2023. We examined the SNPs in genes known to confer or be related to antifungal resistance for individual clades and identified both individual clade-specific and shared SNPs between clades. In total, we found three shared SNPs between Clades I and IV, one between Clades I and III, and zero between Clades III and IV. Although Clades I and IV exhibit greater genetic distance compared to Clades I and III, more antifungal-related SNPs were shared between Clades I and IV than between Clades I and III.

Within each of the three clades, over a dozen candidate antifungal-related SNPs were identified. A few of the mutations such as the Ser639Pro mutation in *FKS1* are known to be associated with echinocandins MIC differences. Interestingly, the Ser639Pro mutation in *FKS1* showed up in both Clades I and IV, with the amino acid Pro associated with elevated echinocandins MICs in both clades. However, most other identified mutations were clade-specific and have not been reported before as being associated with antifungal-susceptibility differences, likely due to the fact that either some of these mutations originated independently among strains and clades or that there was no statistical power to support their identification even if they were present in all clades. Indeed, many genes containing SNPs associated with drug susceptibility differences belonged to the “unknown” function category, largely due to the incomplete annotation of the *C. auris* genome and other *Candida* genomes [18]. Improved annotations of genomes of *Candida* spp., including those of *C. auris* genomes from different clades, could help to identify the specific genes associated with drug resistance. Furthermore, targeted analyses of transcriptome data, real-time quantitative PCR, and/or gene knockouts/knock-ins are needed to confirm the roles of these candidate SNPs in antifungal drug susceptibility differences.

Though many novel SNPs were identified as associated with antifungal-susceptibility differences in all three clades, for several reasons, we believe the numbers we identified represent an underestimate of the true number of genes and genetic variants contributing to drug resistance in *C. auris*. First, for several drugs, we observed small variations in MICs among strains within individual clades, with a biased distribution towards high MICs (Figure 1). Such a distribution pattern makes it difficult to identify SNPs associated with susceptibility differences. Second, SNPs with minor allele frequencies less than 1% were excluded from our analyses. Thus, antifungal-resistant SNP sites with minor allele frequencies less than 1% would not be identified in our study. Another factor is that there are no MIC data for many strains with genome sequence data. Even though 4303 strains have been sequenced, only 387 strains (<10%) have associated drug susceptibility data in the public domain. Consequently, potentially unique mutations in those strains that are associated with drug resistance could not be identified. To increase the power of detection, it is highly recommended that future reports of *C. auris* strains and genomes make their antifungal-susceptibility data publicly available.

While the above limitations can result in underestimates of drug susceptibility-associated genomic variants, several other issues may also influence our results. First, the antifungal-susceptibility profiles reported for the analyzed strains were generated using different methods. They include those of the Clinical and Laboratory Standards Institute (CLSI), the European Committee on Antimicrobial Susceptibility Testing (EUCAST), Sensititre YeastOne panels (SYO), Etest, and the Vitek system. Discrepancies can occur in MIC values when using different approaches. Essential agreement (EA) and categorical agreement (CA) are two indexes used to assess the agreement between different standards. The former refers to the discrepancies in MICs with no more than +/− 2-fold dilutions, while the latter indicates the agreement in the categorization of samples as susceptible or resistant to a specific drug. For Clades I, III, and IV, 60.8% of the strains had their profiles determined using the CLSI method, 3.2% using EUCAST, and 35.4% using the SYO method. Previous studies have shown that the SYO and CLSI methods have high CA and EA consistencies for echinocandins but a low CA value for azoles for *Candida* spp. [49,50,51]. Such (in)consistencies among testing methods might have contributed to the higher number of genomic variants that we identified as associated with susceptibility differences for echinocandins than for azoles. The second limitation is the relatively small sample sizes. Even though there are six genetically distinct clades within *C. auris* with over 4000 genome sequences deposited, we were only able to collect modest sample sizes for three clades, Clades I, III and IV, for this study. With more strains sequenced and their susceptibility data deposited in public domains, a more comprehensive understanding of the antifungal resistance mechanisms in this species could be obtained.

The alternative GWAS analyses using MIC values standardized in log scale found eight SNPs associated with FLU MICs in Clade I. Among these eight SNPs, only one overlapped with our original FLU GWAS in Clade I (where four SNPs were identified, Table 3). Interestingly, one of the new ones, a missense mutation, was originally identified as linked to AMB susceptibility. However, the remaining six were all new. The increased number of SNPs was due to the expanded range of MIC values analyzed here (doubled from 257 mg/L to 512 mg/L). Unfortunately, the alternative analyses for the FLU and 5FC GWASs for Clade III did not yield any meaningful results, likely due to the highly skewed distributions of MICs in the alternative scheme. Given that the MIC values can have a considerable effect on the GWAS analyses, the roles of the identified SNPs in antifungal resistance need to be further validated. Meanwhile, considering the high-level resistance of most strains of *C. auris* to many antifungals, it is important to include a broad range of drug concentrations such as concentrations higher than 256 mg/L for FLU in antifungal-susceptibility testing to obtain an accurate MIC for strains in this species. Such data will improve our understanding of the genetic mechanisms underlying drug resistance in *C. auris* and enhance our ability to track the evolution and spread of drug-resistance among populations of this pathogen across environments [52].

## 5. Conclusions

In this study, we performed GWAS analyses on Clade I, III, and IV strains of *C. auris* with published MIC values and genomic data. For each clade, we examined from eight to nine antifungal drugs and identified a number of antifungal-susceptibility-related SNPs. Specifically, within Clade I, a total of 15 antifungal-susceptibility-related SNPs were identified, with 10 in coding regions and 5 in noncoding regions. In Clade III, 24 SNPs were identified, with 11 in coding regions and 13 in noncoding regions. Clade IV had 13 SNPs, with 10 in coding regions and 3 in noncoding regions. Compared with reference strains, the alternative nucleotides at these SNP sites were shown to have variable impacts on antifungal susceptibilities, with some being associated with elevated MICs, while others associated with reduced MICs. Interestingly, a few nonsynonymous SNPs identified here have been shown to confer antifungal resistance in *Candida* according to previous research, consistent with the relative robustness of our approach. In addition, several mutations in noncoding regions were shown to be located close to genes with known drug transmembrane transport and membrane integrity activities. However, most of the antifungal-susceptibility-related SNPs identified here are novel, and not reported to be associated with drug resistance in previous studies. The roles of these SNPs in antifungal resistance need to be experimentally validated. If confirmed, these genes and their genetic variants could serve as biomarkers for the rapid diagnosis of drug susceptibilities and as targets from which to develop novel drugs.

## Figures and Tables

**Table 1:** Sample distribution and MIC statistics for GWAS analysis across three clades of *C. auris*, categorized by antifungal drugs.

Antifungal	Clade I (152)	Mean (Range)	Clade III (119)	Mean (Range)	Clade IV (99)	Mean (Range)
5FC	107	9.83 (0.03–65)	110	0.12 (0.05–0.5)	1	0.5 (–)
AMB	148	1.89 (0.25–8)	119	0.95 (0.125–2)	96	1.04 (0.25–4)
FLU	126	151.96 (0.5–257)	111	246.66 (8–256)	96	13.20 (1–257)
VOR	147	2.22 (0.008–16)	119	1.53 (0.06–8)	88	0.29 (0.015–8)
ITR	134	1.65 (0.015–16)	115	0.28 (0.06–1)	86	0.19 (0.02–1)
POS	131	0.86 (0.008–8)	114	0.08 (0.015–0.25)	86	0.09 (0.015–0.5)
AFG	141	0.49 (0.015–9)	119	0.41 (0.032–8)	88	0.57 (0.016–4)
CAS	134	1.34 (0.008–16)	118	0.27 (0.06–4)	96	0.35 (0.016–9)
MFG	145	0.44 (0.015–9.0)	116	0.28 (0.06–8.0)	92	0.42 (0.03–9.0)

5FC: 5-fluorocytosine; AMB: amphotericin B; FLU: fluconazole; VOR: voriconazole; ITR: itraconazole; POS: posaconazole; AFG: anidulafungin; CAS: caspofungin; MCF: micafungin. In the column names, the number next to each clade indicates the number of strains containing MICs for any of the nine antifungals. The numbers beneath each clade are the numbers of strains used for individual GWAS analyses after excluding strains which failed MIC standardization.

**Table 2:** *C. auris* Clade-shared SNPs in previously reported antifungal resistance-related genes in the genus *Candida*.

Clade	Gene	Chr	Pos	Corresponding SNP Site in Clade I	REF	ALT	Mutations	REF_n	ALT_n
I	*ERG11*	PEKT02000003.1	833880	PEKT02000003.1_833880	T	C	Lys143Arg	119	59
I	*ERG11*	PEKT02000003.1	833913	PEKT02000003.1_833913	T	A	Tyr132Phe	62	116
I	*FKS1*	PEKT02000002.1	1006624	PEKT02000002.1_1006624	G	T, A	Ser639Phe, Tyr	170	5
I	*FKS1*	PEKT02000002.1	1006625	PEKT02000002.1_1006625	A	G	Ser639Pro	176	2
III	*FKS1*	NW_021640162.1	2137612	PEKT02000002.1_1006624	G	A	Ser639Phe	117	2
IV	*ERG11*	CP043444.1	1566724	PEKT02000003.1_833880	T	C	Lys143Arg	97	2
IV	*ERG11*	CP043444.1	1566757	PEKT02000003.1_833913	A	T	Phe132Tyr	3	96
IV	*FKS1*	CP043443.1	2100709	PEKT02000002.1_1006625	A	G	Ser639Pro	97	2

Chr: scaffold; Pos: position; REF: reference allele; REF_n: count of reference allele; ALT: alternative allele; ALT_n: count of alternative allele; rows in same color indicate the same SNP loci among different clades. Each unique SNP is shaded in a distinct color.

**Table 3:** Significant SNPs associated with antifungal drugs identified in Clade I GWAS.

Drug	SNP ID	MAF	Nobs	FDR Adjusted *p*-Value	Effect	Parameters	Annotation	HGVS.c	HGVS.p	Gene	*Candida albicans* Ortholog
FLU	PEKT02000001.1_897129	0.036	126	3.01 × 10^−8^	−101.826	FarmCPU, PCA:10	intergenic	c.-3672G>T			
	PEKT02000003.1_642734	0.036	126	4.84 × 10^−7^	188.652		intergenic	c.-4186G>A			
	PEKT02000003.1_1045994	0.028	126	6.80 × 10^−5^	201.408		intergenic	c.-4975T>A			
	PEKT02000002.1_75394	0.04	126	4.22 × 10^−3^	65.982		synonymous	c.2097A>G	p.Gln699Gln	*B9J08_000534*	CR_10440
AMB	PEKT02000003.1_514453	0.024	148	1.39 × 10^−6^	−4.813	FarmCPU, PCA:10	synonymous	c.672G>T	p.Ala224Ala	*B9J08_001302*	*BRE1*
	PEKT02000003.1_517475	0.162	148	2.30 × 10^−4^	−1.475		synonymous	c.849C>T	p.His283His	B9J08_001303	*BDF1*
	PEKT02000002.1_906944	0.088	148	1.21 × 10^−3^	1.789		missense	c.1465T>C	p.Ser489Pro	B9J08_000923	SWC4
	PEKT02000002.1_45839	0.014	148	1.22 × 10^−3^	−1.869		missense	c.1028T>C	p.Ile343Thr	B9J08_000517	FET31
CAS	PEKT02000002.1_1006625	0.015	134	1.63 × 10^−18^	7.42	BLINK, PCA:5	missense	c.1915T>C	p.Ser639Pro	*B9J08_000964*	*FKS1*
MFG	PEKT02000002.1_1006624	0.014	145	2.26 × 10^−24^	6.174	BLINK, PCA:3	missense	c.1916C>T	p.Ser639Phe	*B9J08_000964*	*FKS1*
	PEKT02000002.1_1006625	0.014	145	4.51 × 10^−11^	2.514		missense	c.1915T>C	p.Ser639Pro	*B9J08_000964*	*FKS1*
	PEKT02000004.1_9898	0.041	145	2.60 × 10^−5^	0.832		synonymous	c.3261C>T	p.Thr1087Thr	*B9J08_001531*	*IFF4*
	PEKT02000002.1_1006624	0.014	145	1.10 × 10^−26^	6.234	FarmCPU, PCA:3	missense	c.1916C>W	p.Ser639Phe/Tyr	*B9J08_000964*	*FKS1*
	PEKT02000003.1_355435	0.014	145	5.83 × 10^−12^	−2.546		stop_gained	c.1012C>T	p.Gln338 *	*B9J08_001232*	*FCR1*
	PEKT02000004.1_9898	0.041	145	7.37 × 10^−6^	0.848		synonymous	c.3261C>T	p.Thr1087Thr	*B9J08_001531*	*IFF4*
	PEKT02000002.1_623810	0.128	145	4.81 × 10^−3^	−0.534		intergenic	c.4486G>A			
AFG	PEKT02000002.1_1006624	0.014	141	5.53 × 10^−29^	7.5103	BLINK, PCA:8	missense	c.1916C>T	p.Ser639Phe	*B9J08_000964*	*FKS1*
	PEKT02000003.1_355435	0.014	141	1.29 × 10^−15^	−3.6731		stop_gained	c.1012C>T	p.Gln338 *	*B9J08_001232*	*FCR1*
	PEKT02000002.1_804575	0.032	141	4.50 × 10^−7^	1.8354		synonymous	c.369G>A	p.Lys123Lys	*B9J08_000876*	*GWT1*
	PEKT02000001.1_212518	0.035	141	1.98 × 10^−3^	−0.7039		intergenic	c.-471G>A			

Shared SNPs are highlighted in the same colors. MAF: minor allele frequency; nobs: sample size; Effect: the increase in phenotype values due to the increase in genotype values per unit; Parameters: GWAS methods, numbers of principal components as covariates; HGVS: Human Genome Variation Society; HGVS.c: variant using HGVS notation (DNA level); HGVS.p: variant using HGVS notation (Protein level); *: nonsense mutation; Gene: the genes in which the specific coding-region SNPs are located. Each SNP identified by different methods (BLINK and FarmCPU) or associated with susceptibilities to different drugs is highlighted in a distinct color.

**Table 4:** Significant SNPs associated with antifungal-susceptibility differences among strains in Clade III.

Drug	SNP	MAF	Nobs	FDR Adjusted *p*-Value	Effect	Parameters	Annotation	HGVS.c	HGVS.p	Gene	*Candida albicans* Ortholog
FLU	NW_021640166.1_27489	0.04	111	1.05 × 10^−130^	63.924	BLINK, PCA:8	synonymous	4041T>A	Thr1347Thr	*B9J08_004201*	*C4_05790W_A*
	NW_021640163.1_757345	0.03	111	1.44 × 10^−99^	261.051		intergenic	−4102G>T			
	NW_021640164.1_51689	0.49	111	1.44 × 10^−99^	260.855		intergenic	−781C>T			
	NW_021640164.1_51692	0.5	111	8.53 × 10^−99^	261.523		intergenic	−784C>T			
	NW_021640168.1_1173685	0.01	111	3.69 × 10^−53^	111.826		intergenic	−2970A>G			
	NW_021640164.1_773750	0.45	111	1.28 × 10^−49^	−68.521		upstream	−867C>T			
	NW_021640162.1_73156	0.02	111	3.17 × 10^−49^	34.103		synonymous	993C>T	Cys331Cys	*B9J08_000516*	*C6_00490W_A*
	NW_021640165.1_3471968	0.01	111	4.53 × 10^−36^	−22.502		missense	2267G>T	Gly756Val	*B9J08_003726*	*C2_04360W_A*
	NW_021640162.1_147544	0.02	111	2.23 × 10^−26^	21.65		missense	124G>T	Ala42Ser	*B9J08_000558*	*CR_09700W_A*
	NW_021640164.1_37868	0.02	111	1.01 × 10^−10^	20.514		intergenic	−1343C>G			
CAS	NW_021640163.1_1501688	0.02	118	1.80 × 10^−83^	1.922	FarmCPU, PCA:5	synonymous	1245C>T	Cys415Cys	*B9J08_001552*	*orf19.3701*
	NW_021640164.1_366204	0.02	118	4.70 × 10^−12^	0.712		missense	663A>T	Arg221Ser	*B9J08_002399*	*SUP35*
	NW_021640168.1_1068005	0.01	118	0.0096	0.16		stop_gained	879C>A	Tyr293 *	*B9J08_005386*	*C6_01620W_A*
	NW_021640163.1_1501688	0.02	118	5.38 × 10^−82^	1.896	BLINK, PCA:5	synonymous	1245C>T	Cys415Cys	*B9J08_001552*	*orf19.3701*
	NW_021640163.1_505104	0.02	118	2.58 × 10^−06^	0.591		missense	706T>C	Tyr236His	*B9J08_001308*	*EIP1*
	NW_021640164.1_51605	0.32	118	0.0138	−0.053		intergenic	−697C>T			
MFG	NW_021640162.1_404574	0.02	116	2.86 × 10^−124^	−3.939	FarmCPU, PCA:5	intergenic	−4910C>G			
	NW_021640162.1_1734842	0.11	116	0.0045	0.069		intergenic	−1408A>G			
	NW_021640165.1_1713220	0.02	116	0.00036	0.111		missense	6345T>G	Asp2115Glu	*B9J08_002877*	*C4_06130W_A*
	NW_021640162.1_404574	0.02	116	5.63 × 10^−124^	−3.942	BLINK, PCA:5	intergenic	−4910C>G			
	NW_021640162.1_564168	0.03	116	0.0019	0.145		synonymous	1203C>T	Pro401Pro	*B9J08_000751*	*CR_01410C_A*
	NW_021640165.1_1713220	0.02	116	0.0029	0.101		missense	6345T>G	Asp2115Glu	*B9J08_002877*	*C4_06130W_A*
AFG	NW_021640162.1_692140	0.02	119	1.15 × 10^−11^	1.418	FarmCPU, PCA:5	missense	520A>T	Ser174Cys	*B9J08_000805*	*ZCF19*
	NW_021640163.1_1501688	0.02	119	1.00 × 10^−100^	3.877		synonymous	1245C>T	Cys415Cys	*B9J08_001552*	*orf19.3701*
	NW_021640168.1_217077	0.03	119	0.0055	0.298		intergenic	−4324G>A			
	NW_021640165.1_1276391	0.05	119	2.29 × 10^−06^	0.381		intergenic	−2259A>T			
	NW_021640163.1_1501688	0.02	119	1.74 × 10^−98^	3.87	BLINK, PCA:5	synonymous	1245C>T	Cys415Cys	*B9J08_001552*	*orf19.3701*
	NW_021640162.1_1360735	0.18	119	0.0024	−0.393		intron	−2009G>A			
	NW_021640166.1_397189	0.03	119	0.00312	0.244		intergenic	−1210T>A			

Shared SNPs are highlighted in the same colors. MAF: minor allele frequency; nobs: sample size; Effect: the increase in phenotype values due to the increase in genotype values per unit; Parameters: GWAS methods, numbers of principal components as covariates; HGVS: Human Genome Variation Society; HGVS.c: variant using HGVS notation (DNA level); HGVS.p: variant using HGVS notation (protein level); *: nonsense mutation; Gene: the genes in which the specific coding-region SNPs are located. Each SNP identified by different methods (BLINK and FarmCPU) or associated with susceptibilities to different drugs is highlighted in a distinct color.

**Table 5:** Significant SNPs associated with antifungal-susceptibility differences among strains in Clade IV.

Drug	SNP	MAF	Nobs	FDR Adjusted *p*-Value	Effect	Parameters	Annotation	HGVS.c	HGVS.p	Gene	*Candida albicans* Ortholog
VOR	CP043444.1_1566757	0.023	88	7.66 × 10^−52^	−1.76	FarmCPU, PCA:10	missense	c.395T>A	p.Phe132Tyr	*B9J08_001448*	*ERG11*
	CP043442.1_1367963	0.011	88	2.35 × 10^−15^	0.367		missense	c.4328C>T	p.Ser1443Leu	*B9J08_003473*	*PEP1*
	CP043444.1_1393470	0.023	88	0.00041	0.111		missense	c.116A>T	p.Asn39Ile	*B9J08_001368*	*DAK2*
CAS	CP043442.1_1778999	0.01	96	7.77 × 10^−15^	0.464	FarmCPU, PCA:12	missense	c.3292T>C	p.Phe1098Leu	*B9J08_003281*	*RIM15*
	CP043443.1_1796780	0.094	96	1.50 × 10^−5^	0.228		intergenic	c.-4835A>G			
	CP043443.1_2100709	0.021	96	1.04 × 10^−32^	4.725		missense	c.1915T>C	p.Ser639Pro	*B9J08_000964*	*FKS1*
	CP043444.1_498630	0.141	96	0.00044	0.144		stop_gained	c.964A>T	p.Arg322 *	*B9J08_002136*	*WOR2*
	CP043446.1_343550	0.083	96	9.95 × 10^−5^	0.093		missense	c.488C>T	p.Pro163Leu	*B9J08_004612*	*EDC3*
	CP043443.1_2100709	0.021	96	2.38 × 10^−34^	4.551	BLINK, PCA:12	missense	c.1915T>C	p.Ser639Pro	*B9J08_000964*	*FKS1*
	CP043443.1_1645903	0.01	96	2.33 × 10^−14^	−0.464		synonymous	c.600G>A	p.Glu200Glu	*B9J08_000760*	*C4_01930*
	CP043444.1_498630	0.141	96	9.68 × 10^−6^	0.162		stop_gained	c.964A>T	p.Arg322 *	*B9J08_002136*	*WOR2*
	CP043443.1_1796780	0.094	96	0.000367	0.206		intergenic	c.-4835A>G			
	CP043442.1_182935	0.083	96	0.00625	−0.077		intergenic	c.-4482C>T			
MFG	CP043442.1_514989	0.011	92	4.55 × 10^−53^	−4.452	BLINK, PCA:5	missense	c.400G>C	p.Gly134Arg	*B9J08_002701*	*C7_04260*
	CP043442.1_613136	0.011	92	1.05 × 10^−13^	−0.797		missense	c.590C>T	p.Pro197Leu	*B9J08_003827*	*C2_10320*
	CP043444.1_498630	0.147	92	3.14 × 10^−6^	0.321		stop_gained	c.964A>T	p.Arg322 *	*B9J08_002136*	*WOR2*

Shared SNPs are highlighted in the same colors. MAF: minor allele frequency; nobs: sample size; Effect: the increase in phenotype values due to the increase in genotype values per unit; Parameters: GWAS methods, numbers of principal components as covariates; HGVS: Human Genome Variation Society; HGVS.c: variant using HGVS notation (DNA level); HGVS.p: variant using HGVS notation (Protein level); *: nonsense mutation; Gene: the genes in which the specific coding-region SNPs are located. Each SNP identified by different methods (BLINK and FarmCPU) or associated with susceptibilities to different drugs is highlighted in a distinct color.

**Table 6:** Details of noncoding SNPs in linkage disequilibrium with other significant SNPs.

Clade	Drug	Noncoding SNP	Significant Linked SNPs	r2	FDR Adjusted *p*-Value for Linked SNPs
I	FLU	PEKT02000001.1_897129	PEKT02000003.1_1045994	0.057	0.0209
		PEKT02000003.1_642734	PEKT02000002.1_1006624	0.167	3.68 × 10^−8^
		PEKT02000003.1_1045994	PEKT02000001.1_897129	0.057	0.0209
	MFG	PEKT02000002.1_623810	-	-	-
	AFG	PEKT02000001.1_212518	-	-	-
III	FLU	NW_021640163.1_757345	-	-	-
		NW_021640164.1_51689	-	-	-
		NW_021640164.1_51692	-	-	-
		NW_021640168.1_1173685	-	-	-
		NW_021640164.1_773750	-	-	-
		NW_021640164.1_37868	-	-	-
	CAS	NW_021640164.1_51605	NW_021640165.1_1276391	0.135	0.0119
	MFG	NW_021640162.1_404574	-	-	-
		NW_021640162.1_1734842	-	-	-
	AFG	NW_021640168.1_217077	-	-	-
		NW_021640165.1_1276391	NW_021640164.1_51605	0.135	0.0119
		NW_021640162.1_1360735	-	-	-
		NW_021640166.1_397189	-	-	-
IV	CAS	CP043443.1_1796780	-	-	-
		CP043442.1_182935	-	-	-

r2: linkage disequilibrium measure. SNPs showing reciprocal significant associations are highlighted in the same color, red and green respectively here.

## Data Availability

Data are contained within the article and Supplementary Materials.

## Supplementary Materials

The following supporting information can be downloaded at: https://www.mdpi.com/article/10.3390/jof10010086/s1, Table S1. Details of samples used in this study; Table S2. SNPs in reported antifungal resistance genes; Table S3. Genes adjacent to the 16 noncoding SNPs independent of linkage to coding SNPs; Table S4. GO enrichment analysis genes flanking the 19 noncoding SNPs. Table S5. Significant SNPs associated with FLU susceptibility in Clade I identified by additional GWAS analysis. Figure S1. QQ plot and Manhattan plot showing FLU associated SNPs in Clade I identified by additional GWAS analysis.
