# Supplementary figures and images for: Associations between Genomic Variants and Antifungal Susceptibilities in the Archived Global Candida auris Population
Source: J Fungi (Basel). 2024 Jan 22;10(1):86. doi: 10.3390/jof10010086 (PMC10821368; doi:10.3390/jof10010086)

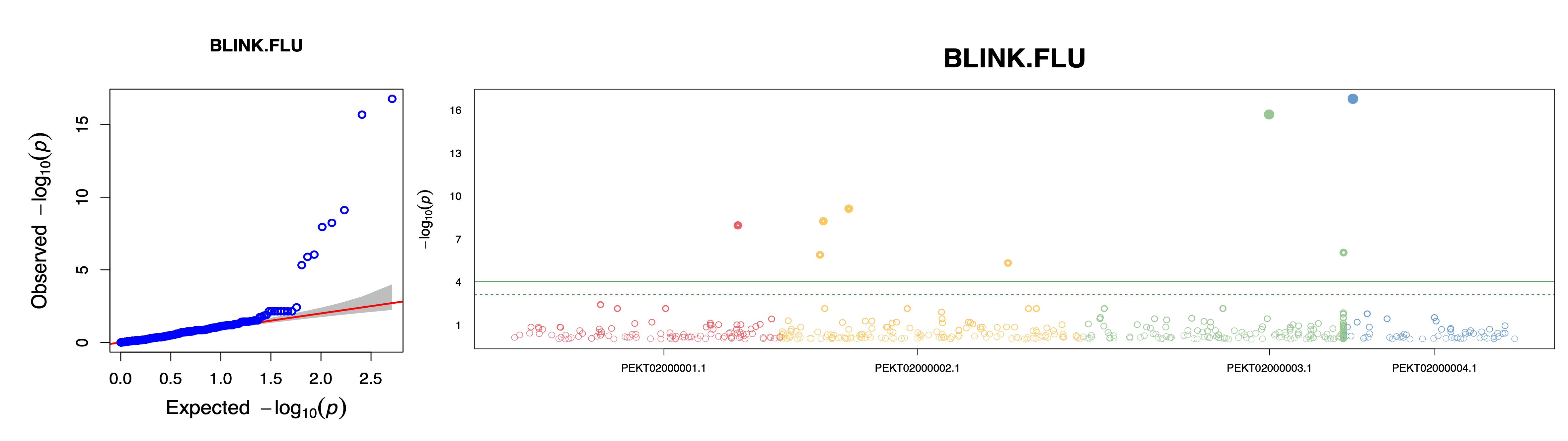

Supplement: Supplementary file 1 [file jof-10-00086-s001.zip › Figure S1.png]
